# Early prediction of the impact of public health policies on obesity and lifetime risk of type 2 diabetes: A modelling approach

**DOI:** 10.1371/journal.pone.0301463

**Published:** 2024-03-28

**Authors:** Pierre Bauvin, Claire Delacôte, Line Carolle Ntandja Wandji, Guillaume Lassailly, Violeta Raverdy, François Pattou, Sylvie Deuffic-Burban, Philippe Mathurin

**Affiliations:** 1 Inserm, CHU Lille, U1286 –INFINITE–Institute for Translational Research in Inflammation, Université de Lille, Lille, France; 2 Services Maladies de l’Appareil Digestif, Hôpital Claude Huriez, CHRU Lille, Lille, France; 3 Inserm, CHU Lille, U1190—EGID, Univ. Lille, Lille, France; 4 Inserm IAME, Université Paris Cité and Université Sorbonne Paris Nord, Paris, France; Institute of Tropical Medicine: Instituut voor Tropische Geneeskunde, BELGIUM

## Abstract

**Objective:**

Help public health decision-making requires a better understanding of the dynamics of obesity and type 2 diabetes and an assessement of different strategies to decrease their burdens.

**Methods:**

Based on 97,848 individual data, collected in the French Health, Health Care and Insurance Survey over 1998–2014, a Markov model was developed to describe the progression of being overweight to obesity, and the onset of type 2 diabetes. This model traces and predicts 2022–2027 burdens of obesity and type 2 diabetes, and lifetime risk of diabetes, according to different scenarios aiming at minimum to stabilize obesity at 5 years.

**Results:**

Estimated risks of type 2 diabetes increase from 0.09% (normal weight) to 1.56% (obesity II-III). Compared to the before 1995 period, progression risks are estimated to have nearly doubled for obesity and tripled for type 2 diabetes. Consequently, over 2022–2027, the prevalence of obesity and type 2 diabetes will continue to increase from 17.3% to 18.2% and from 7.3% to 8.1%, respectively. Scenarios statibilizing obesity would require a 22%-decrease in the probability of move up (scenario 1) or a 33%-increase in the probability of move down (scenario 2) one BMI class. However, this stabilization will not affect the increase of diabetes prevalence whereas lifetime risk of diabetes would decrease (30.9% to 27.0%). Combining both scenarios would decrease obesity by 9.9%. Only the prevalence of obesity III shows early change able to predict the outcome of a strategy: for example, 6.7%-decrease at one year, 13.3%-decrease at two years with scenario 1 stabilizing obesity at 5 years.

**Conclusions:**

Prevalences of obesity and type 2 diabetes will still increase over the next 5 years. Stabilizing obesity may decrease lifetime risks of type 2 diabetes without affecting its short-term prevalence. Our study highlights that, to early assess the effectiveness of their program, public health policy makers should rely on the change in prevalence of obesity III.

## Introduction

Overweight and obesity are major public health challenges because of their steadily rising prevalence worldwide in all segments of society [1]. The development of obesity is mainly related to the consumption of increasingly high energy food products and a decrease in daily physical activity. The prevalence of obesity varies in populations according to socio-economic factors, cultural environment, ethnicity, and genetic and epigenetic mechanisms [2].

Obesity has consequences to health such as type 2 diabetes, cardiovascular disease and cancer [3]. Type 2 diabetes is an important cause of complications and deaths worldwide, affecting approximately 462 million individuals in 2017 [4]. By 2030 it may become the 7th cause of death worldwide causing 3.0% of total deaths [5]. Most patients with type 2 diabetes also have obesity, and the rising incidence of type 2 diabetes is explained, at least in part, by the relationship between obesity and hyperinsulinemia, due to the response of the pancreas to systemic insulin resistance [6]. Nevertheless, more insight is still needed to clarify the mechanisms linking the two conditions that could help the development of prevention strategies [6].

National and international health initiatives have been created in response to the obesity and type 2 diabetes epidemics. World Health Organization (WHO) adopted a comprehensive global monitoring framework for the prevention and control of noncommunicable diseases. The prevalence of the risk factors of obesity and diabetes can be measured within this framework as well as their progress over time. In France, the prevalence of obesity has nearly doubled in the past 15 years [7, 8] leading health agencies to launch The National Nutritional Health Program (NNHP) to reduce obesity by 15% and stabilize the prevalence of overweight adults before 2023. It is important to determine which strategies are needed to make this program a success, for example reducing the incidence of move up one BMI class versus increasing the incidence of move down one BMI class, and the consequences of these approaches on the incidence of type 2 diabetes. At present decision-makers only have indicators on the prevalence of population with overweight and obesity, which requires a long period to show changes. However, early markers are needed to evaluate public health policies to justify pursuing or interrupting health policy if they are considered to be ineffective.

Mathematical modelling is a method of choice to predict disease progression and determine the extent of modification needed in the parameters. In particular, Markov model, extensively used in medical decision making [9, 10] may also be used to estimate the incidence and lifetime risk of a disease, its trends over time as well as in relation to age and sex, essential indicators to better understand the obesity epidemic and defined the most effective public health policies [11]. To accurately build the model, the availability of a large database of individual observations on BMI and the presence or not of diabetes over time, such as the French Health, Health Care and Insurance Survey (ESPS), is a strength.

To help experts make decisions on public health policies, the present study 1/ quantifies the progression of being overweight, obesity and type 2 diabetes in the general population, 2/ estimates the lifetime risk of diabetes, and 3/ assesses the impact of different strategies to decrease the burden of being overweight, obesity and diabetes.

## Methods

### Overview

A Markov model was developed to describe the progression of being overweight to obesity, and the onset of type 2 diabetes. This model was based on individual data from health surveys representative of the French population conducted every two years between 1998 and 2014 in individuals aged 15 and over. The annual risk of obesity, and developing type 2 diabetes, was quantified in the first step as well as the effect of covariates on these risks. In the second step, the model traced and predicted the prevalence of obesity and type 2 diabetes over time, and evaluated different strategies to reduce their burden. Finally, the model assessed the cumulative risk of developing type 2 diabetes throughout life.

### Markov model

**Fig 1:**
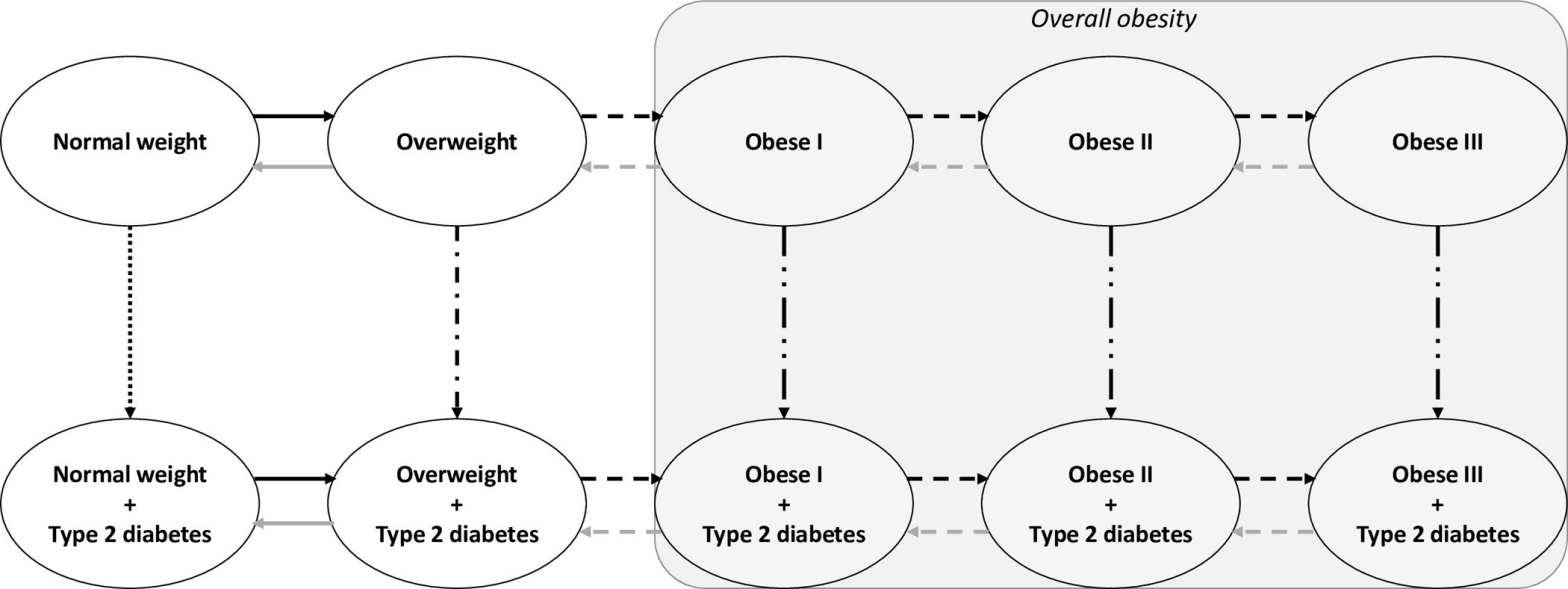
Markov model states and transition.

The progression from being overweight to obesity was modelled based on the usual BMI categories: normal weight (BMI < 25 kg/m^2^), overweight (25 ≤ BMI < 30 kg/m^2^), obesity class I (30 ≤ BMI < 35 kg/m^2^), obesity class II (35 ≤ BMI < 40 kg/m^2^), and obesity class III (BMI ≥ 40 kg/m^2^). The progression from each BMI category to type 2 diabetes was also characterized resulting in 10 health states (Fig 1). In this model, there may be a direct transition from one BMI category to the next or to a previous category, as well as from non-diabetes to diabetes in each BMI category. On the other hand, regression from diabetes to non-diabetes was not included because long-term sustained remission is a marginal event in the general population [12–16].

A different type of arrow indicates a different baseline transition rate to be estimated. Black arrows refers to BMI categories progression and type 2 diabetes onset; grey arrows refers to BMI categories regression.

The effects of potential covariates on these transitions were evaluated (more details are provided further on). We hypothesized that all individuals had a normal weight without type 2 diabetes at the start of the simulations. According to the International Obesity Task Force, overweight and obesity cannot be defined before the age of 2 [17]. Consequently, we considered that overweight and obesity may start from 3 years of age (start of the simulations). We assessed the progression of BMI category and/or onset of type 2 diabetes up to the age of questionnaire answer for each individual.

### Data

To develop the model, we used individual data from the French Health, Health Care and Insurance Survey (ESPS) [18]. This survey on health and the use of healthcare resources was performed every two years from 1998 to 2014 using a self-administered health questionnaire based on a random sample of around 8,000 households among all health insurance beneficiaries aged 15 or older, in metropolitan France (French overseas territories excluded). Our study included only participants with information on weight, height and diabetes. A total of 97,948 individuals were included.

The type 2 diabetes status in a given year was determined from either the response to the specific question on the presence of diabetes or from the list of chronic diseases declared by the respondent. Because according to the National Institute of Diabetes and Digestive and Kidney Diseases, type 2 diabetes most often develops in people over age 45, all individuals under the age of 40 and with normal weight who declared having diabetes were considered to have diabetes from autoimmune or genetic causes and thus, were considered without type 2 diabetes (n = 109, 0.1%) and classified as normal weight. Moreover, our dataset shows no type 2 diabetes before the age of 25 among individuals with overweight or obesity.

To validate externally the developed model, we used published data from the 2020 Obepi-Roche Study launched by the League against Obesity (“Ligue contre l’Obésité”, LCO 2020 Obepi-Roche) [19]. These data contained notably prevalence of overall obesity by age and diabetes (without distinction between mellitus and type 2 diabetes) by class of BMI in 2020, a distant year from the ESPS surveys. Previous Obepi-Roche studies performed every 3 years between 1997 and 2012 conducted to similar prevalences of obesity than observed in ESPS surveys, but access to individual raw data was easier for ESPS [7].

### Baseline trasition rates and covariates

The baseline transition rates represent the risk of moving from one state to another for the reference categories of each covariate: sex (reference = male); age (under 25, 25 to 49, and 50 and over as the reference category) and calendar period (before 1995 as the reference category, 1995 to 2004, and 2005 to 2014). Covariates likely to be associated with the progression from one BMI category to another and with the onset of type 2 diabetes were incorporated into the model through the proportional hazards assumption.

We could not consider all baseline transition rates and covariates effect as potentially different and we had make choices. Indeed, the developed model must be identifiable and parsimonious. Preliminary analyses showed that the fit of the model to the data was improved when we consider an identical progression rate from being overweight toward obesity I, from obesity I to obesity II and from obesity II to obesity III, and a possible identical effect of a given covariate on this baseline rate. Similarly, regression rates from obesity I to becoming overweight and from one class of obesity to the previous one were assumed identical, as well as the incidence of type 2 diabetes in obese II and III. We also assume an identical progression rate through BMI categories with and without type 2 diabetes, as there is no data suggesting type 2 diabetes as an independent risk factor on the change in BMI category. The effect of a given covariate on any baseline regression rates (i.e., from overweight to normal weight, from obesity I to overweight, or from one class of obesity to the previous one) was assumed to be identical. For the other transition rates and covariates effects, no assumptions were considered.

### Procedure

#### Estimation

In the first step, the baseline transition rates, the effects of the covariates, and their 95% confidence intervals (95% CI) were estimated by fitting the developed Markov model (presented in Fig 1) to the data using the maximum likelihood estimates method [20]. We considered that the individuals could progress in BMI category from the age of 3, while the risk of developing type 2 diabetes was considered to start at age 25 for individuals with overweight or obesity, and at age 40 for individuals with normal weight.

#### Validation

In the second step, in order to cover all birth cohorts involved in the ESPS data, the model simulates all birth cohorts from 1895 to the present, through the BMI categories and type 2 diabetes status taking into account the risk of overall mortality. The latter risk was calculated from mortality tables of the French general population and the association of being overweight, obesity and type 2 diabetes with higher all-cause mortality (S1 Appendix) [21–23]. The birth cohort sizes were extracted from national population tables [21].

This second step allows external validation of the model with the 2020 Obepi-Roche Study. The prevalence of obesity and type 2 diabetes in 2020 predicted by the model and observed in the Obepi-Roche Study were compared.

#### Predictions

In the last step, we projected different scenarios of the future burden of obesity (overall, and separately by class) and type 2 diabetes. All scenarios assumed the trend from 2015 to 2022 was similar to that in the last observed period (2005–2014). In the status quo scenario, this trend continued until 2027.

As the French National Nutritional Health Program (NNHP) has set as the goal of reducing by 15% the prevalence of obesity [24], we assessed wether this objective was achievable. We therefore estimated the magnitude of the decrease in the probability of move up one BMI class or the magnitude of the increase in the probability of move down one BMI class, alone or combined, to reach this objective.

Then, we defined a less ambitious objective, which could considered as a public health success if, at least, a stabilization of the prevalence of overall obesity would be observed in 2027 compared to 2022. Two scenarios were built up to attain this stabilization: Scenario 1 simulates a decrease in the probability of move up one BMI category, i.e the decrease in the progression rates from one BMI category to the next; Scenario 2 simulates an increase in the probability of move down in individuals with overweight or obesity, i.e the increase in the regression rates from one BMI category to the previous one.

The impact of a combination of scenarios 1 and 2 was evaluated too (Scenario 3).

Finally, we projected the lifetime cumulative risk of developing type 2 diabetes for different individuals in 2022, according to their BMI category in 2022 and to the three scenarios previously described. That cumulative risk took into account the risk of death at all age, as well as the probability of move up or move down one BMI category.

### Ethics statement

Data are owned by a third organization, IRDES (French Institute for Research and Information in Health Economics), a French governmental institute. These data are subject to national data protection laws and restrictions imposed by the ethics committee to ensure data privacy of the study participants. The access to official anonymized research production files (FPR) was authorized through an individual project agreement at diffusion.adisp@cnrs.fr(application number: 19053).

### Software and sharing code

Analyses were performed with R-software (https://www.R-project.org/) version 3.6.1 and the msm package [20]. This work was performed using high performance computing (HPC) resources from the “Mésocentre” computing center of University of Lille (https://hpc.univ-lille.fr/). Zenodo was used to assign a DOI to share the code used for model fitting: 10.5281/zenodo.10024155.

## Results

### Participants characteristics

The ESPS data obtained from the 9 surveys (every 2 years from 1998 to 2014) represent a total of 97,948 participants aged over 15 years-old. The mean age of participants is 44.7 years (± 18.7), with 51.5% of men (S1 Table). The prevalence of individuals with BMI ≥ 25 kg/m2 (overweight or obesity) in the ESPS cohort was 39.5%, and increased from 34.6% in 1998 to 46.5% in 2014. The prevalence of type 2 diabetes was 3.8%, and increased from 2.0% in 2002 to 6.3% in 2014.

### Validation

**Fig 2:**
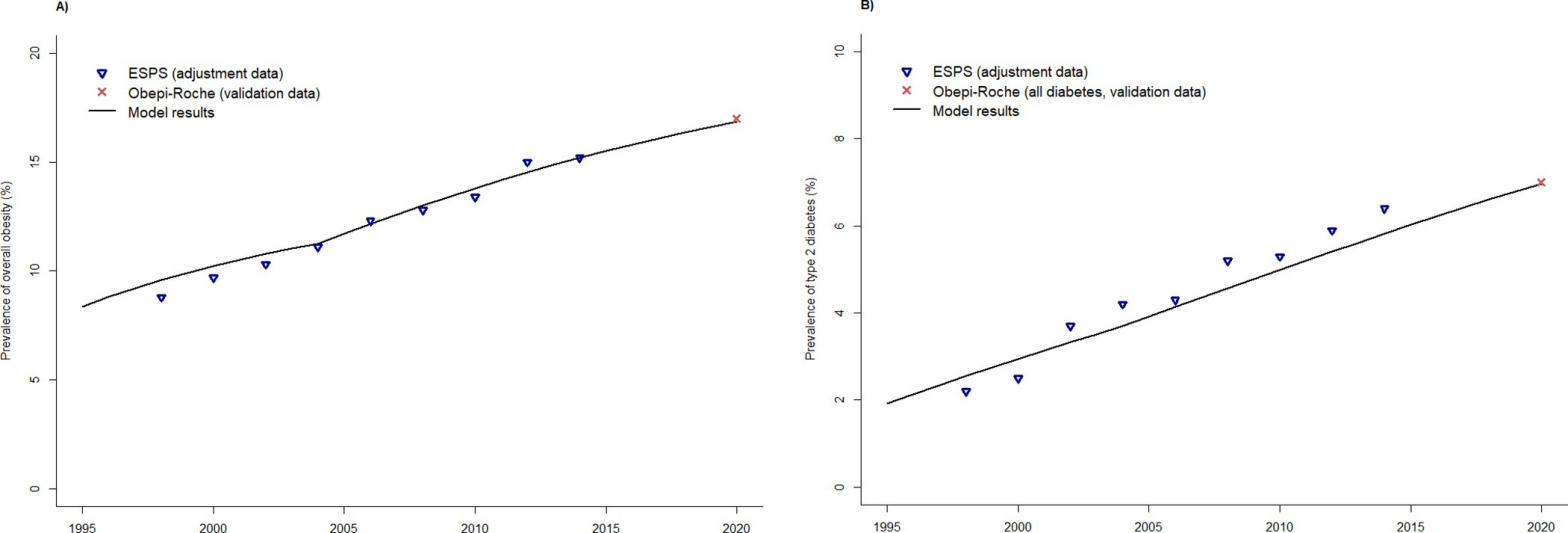
Validation of the model, according to calendar years, in terms of A) prevalence of overall obesity, B) prevalence of type 2 diabetes.

The model fits the observed ESPS data well according to year for obesity (Fig 2A) and type 2 diabetes (Fig 2B).

**Fig 3:**
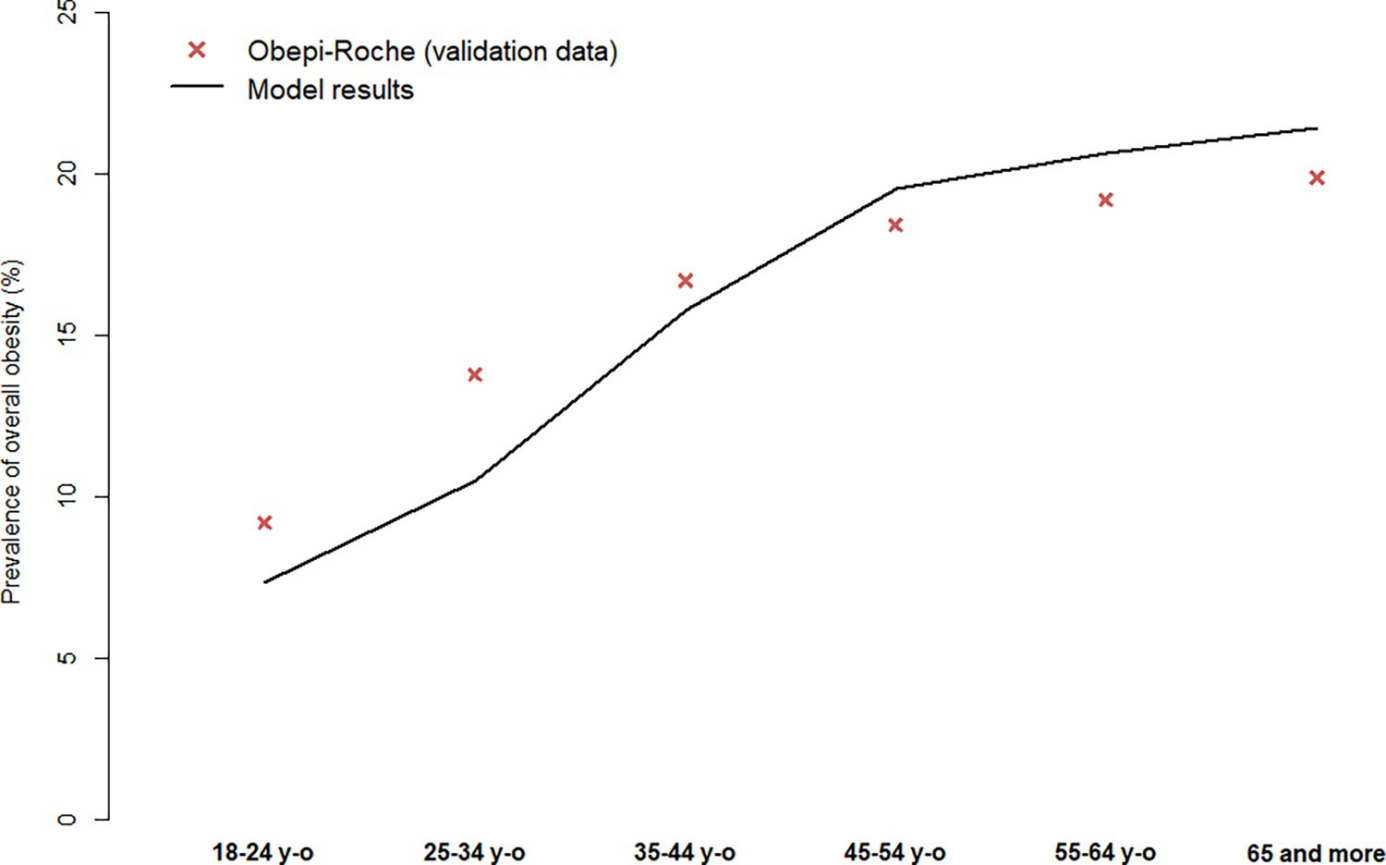
Validation of the model in terms of prevalence of overall obesity according to age group in 2020.

In 2020, the model predicts a prevalence of obesity among ≥ 18 years-old of 16.9%, compared to 17.0% in the Obepi-Roche Study (Fig 2A). In addition, the predicted prevalence of obesity by age class in 2020 was closed to that observed in the Obepi-Roche Study (Fig 3).

**Fig 4:**
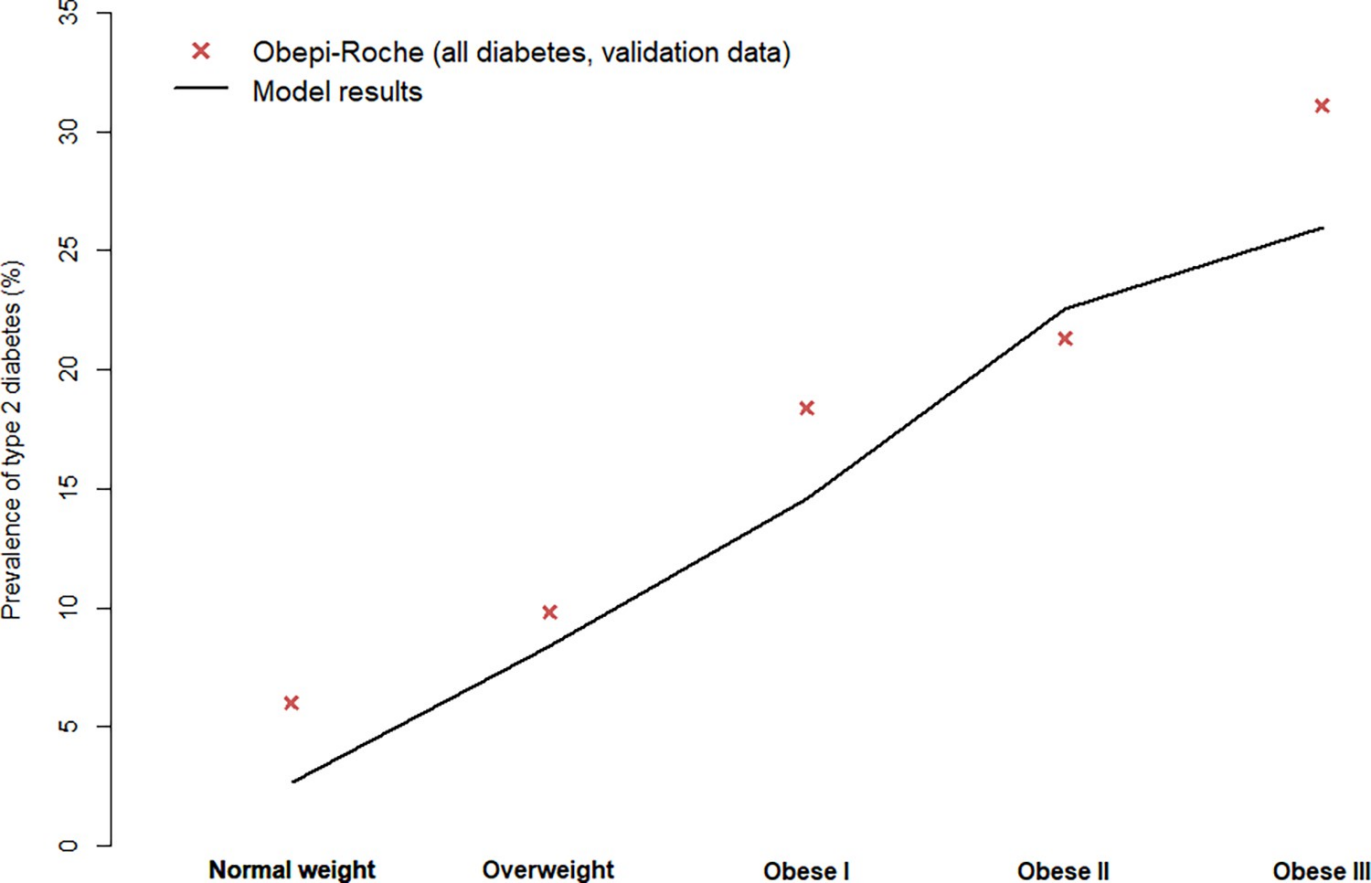
Validation of the model in terms of prevalence of type 2 diabetes according to BMI category in 2020.

Moreover, the prevalence of type 2 diabetes increased when the BMI category increased. According to the model, in 2020, this prevalence varied from 2.8% in normal weight to 26.0% in obese III individuals. In the 2020 Obepi-Roche, the prevalence of diabetes by BMI category was a little higher, from 6.0% to 31.1%, but refers to type 1 and 2 diabetes (Fig 4).

The 2020 Obepi-Roche Study thus confirms external validation of the model, for both outcomes (prevalence of obesity and type 2 diabetes).

### Transition rates and covariates effects

Transition rates towards becoming overweight, obesity and type 2 diabetes are presented in Table 1. The estimated baseline transition rates correspond to men, over 50 years old, during the period before 1995, and the covariate effects are expressed according to these baseline rates. For example, during the period before 1995, men over the age of 50 had a 4.80% [95% CI: 4.48% - 5.15%] rate of progressing from a normal weight to overweight, and 1.25% [95% CI: 1.15% - 1.35%] from being overweight to obesity I, from obesity I to obesity II and from obesity II to obesity III.

**Table 1:** Estimated baseline transition rates and hazard ratios of the covariates impacting these baseline transition rates. Baseline transition rates correspond to the reference, i.e. men, aged ≥ 50 years-old, during the “before 1995” period.

	Parameters	95% confidence intervals
**Baseline transition rates**		
BMI categories progression		
From normal weight to overweight	4.80%	4.48%-5.15%
From overweight to obese I, from obese I to obese II, and from obese II to obese III	1.25%	1.15%-1.35%
BMI categories regression*		
From overweight to normal weight	2.71%	2.44%-3.01%
From obesity I to overweight, from obesity II to obesity I, and from obesity III to obesity II	4.91%	4.36%-5.52%
Type 2 diabetes onset		
For normal weight	0.09%	0.08%-0.10%
For overweight	0.33%	0.31%-0.36%
For obese I	0.81%	0.73%-0.90%
For obese II and III	1.56%	1.39%-1.76%
**Hazard ratios of the covariates**		
For normal weight to overweight		
Female sex	0.72	0.70–0.74
Period category (before 1995 as reference)		
1995–2004	1.11	1.06–1.16
2005–2014	1.43	1.35–1.52
Age category (≥ 50 years-old as reference)		
< 25 years-old	0.24	0.22–0.26
25 to 49 years-old	0.87	0.82–0.92
For overweight to obesity I, for obesity I to obesity II, for obesity II to obesity III		
Female sex	2.00	1.93–2.07
Period category (before 1995 as reference)		
1995–2004	1.92	1.81–2.02
2005–2014	2.38	2.22–2.54
Age category (≥ 50 years-old as reference)		
< 25 years-old	1.48	1.37–1.61
25 to 49 years-old	1.36	1.28–1.44
For BMI categories regression		
Female sex	1.69	1.60–1.79
Period category (before 1995 as reference)		
1995–2004	1.20	1.10–1.31
2005–2014	1.25	1.13–1.39
Age category (≥ 50 years-old as reference)		
< 25 years-old	0.67	0.52–0.87
25 to 49 years-old	1.17	1.07–1.27
For type 2 diabetes onset		
Female sex	0.81	0.77–0.85
Period category (before 1995 as reference)		
1995–2004	2.89	2.66–3.14
2005–2014	3.12	2.84–3.44
Age category (≥ 50 years-old as reference)†		
< 50 years-old	0.24	0.22–0.25

*The parameters indicate the rate of regression from one BMI class to another, but should not be interpreted independently of other progression rates, as progression and regression are competitive events

†Age effect on the development of type 2 diabetes is assumed to be the same for all BMI categories but the age at onset is different: 40 y-o for normal weight individuals, and 25 y-o for individuals with overweight or obesity

The estimated transition rates to type 2 diabetes increase according to BMI category: from 0.09% [95% CI: 0.08% - 0.10%] in normal weight individuals, to 1.56% [95% CI: 1.39% - 1.76%] in obesity II or III (Table 1).

The estimated risks of becoming overweight and obese increase over time: by 1.11 from 1995 to 2004 [95% CI: 1.06–1.16] and by 1.43 from 2005 to 2014 [95% CI: 1.35% - 1.52] for becoming overweight, and by 1.92 [95% CI: 1.81–2.02] and 2.38 [95% CI: 2.22–2.54], respectively, for all categories of obesity, compared to the period before 1995 (Table 1). The estimated risk of type 2 diabetes also increased over time: by 2.89 from 1995 to 2004 [95% CI: 2.66–3.14] and 3.12 from 2005 to 2014 [95% CI: 2.84–3.44], compared to the period before 1995 (Table 1).

Women are at lower risk of becoming overweight (HR = 0.72 [95% CI: 0.70–0.74]) but at higher risk of obesity (HR = 2.00 [95% CI: 1.93–2.07]) compared to men (Table 1).

Younger age was shown to be protective of becoming overweight (HR = 0.24 [95% CI: 0.22–0.26] for < 25 years-old versus ≥ 50 years-old). On the other hand, once a participant became overweight, the risk of obesity was higher at younger age: a 1.48 higher risk [95% CI: 1.37–1.61] for < 25, and 1.36 higher risk [95%CI: 1.28–1.44] for 25–49, versus ≥ 50 years-old (Table 1).

The regression of one BMI category was found to be more probable in women (HR = 1.69 [95% CI: 1.60–1.79] compared to men) and for individuals aged 25–49 years-old (HR = 1.17 [95% CI: 1.07–1.27] versus ≥ 50 years-old) but not in those < 25 years old (HR = 0.67 [95% CI: 0.52–0.87] versus ≥ 50 years-old) (Table 1).

Finally, female sex and younger age were found to be protective of the onset of type 2 diabetes (HR = 0.81 [95%CI: 0.77–0.85] for women, HR = 0.24 [95%CI: 0.22–0.25] for age < 50) (Table 1).

### Model’s predictions of being overweight and obesity over 2022–2027

**Fig 5:**
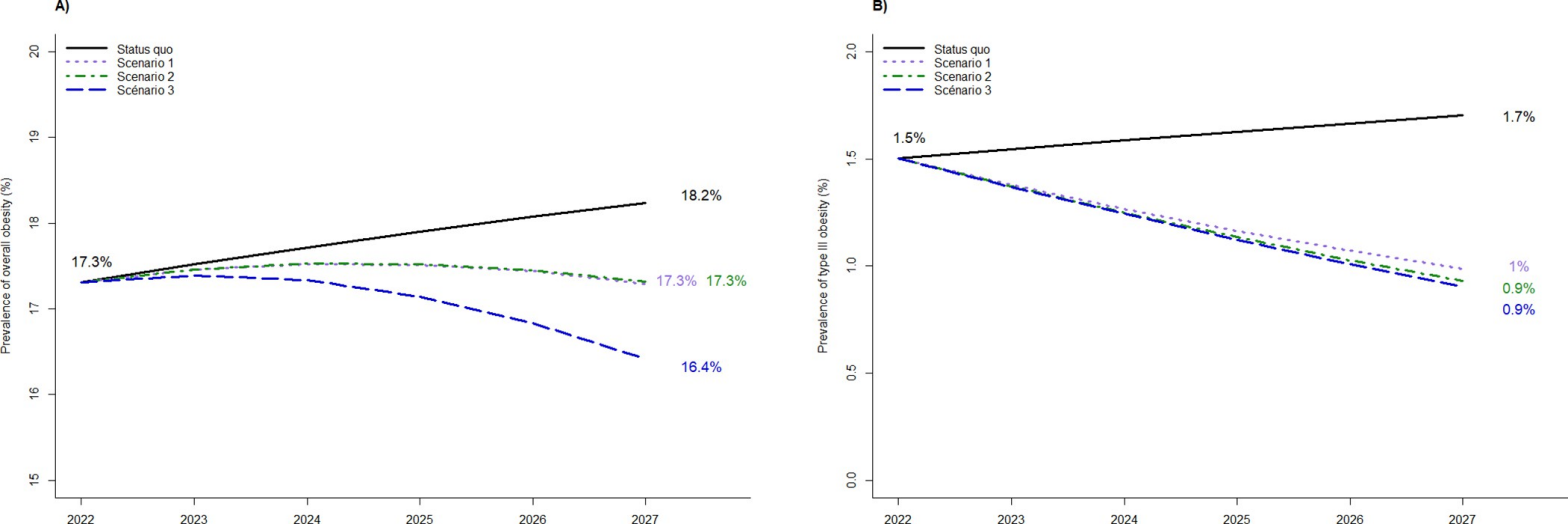
Projection over 2022–2027 of A) overall obesity prevalence, and B) type III obesity prevalence, according to scenarios. Scenarios 1 and 2 were built up to obtain a stabilization of the overall obesity prevalence: scenario 1 corresponds to a 22% decrease in the probability of move up one BMI class, and scenario 2 corresponds to a 33% increase in the probability of move down one BMI class. Scenario 3 corresponds to the combination of scenarios 1 and 2.

**Fig 6:**
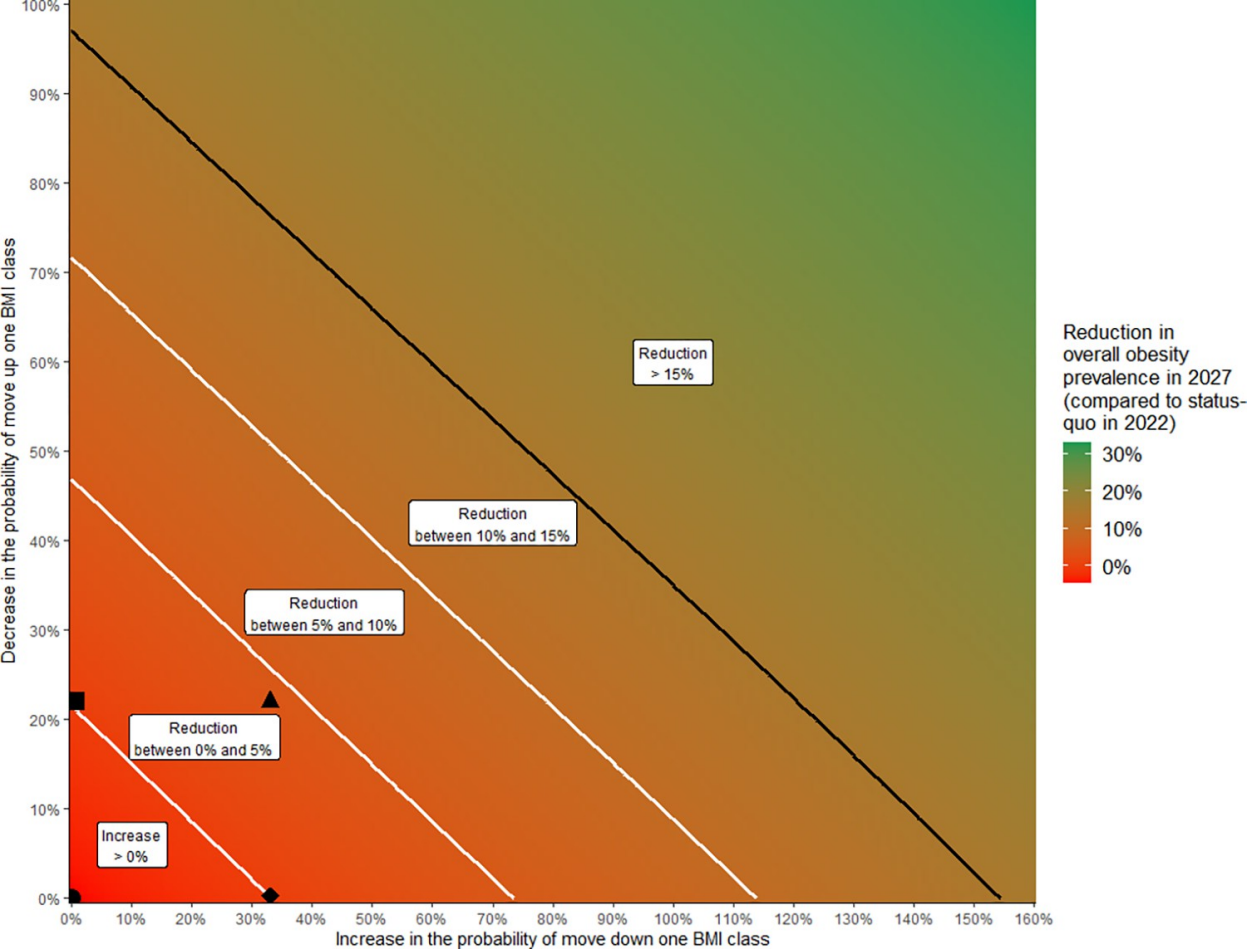
Prevalence reduction of overall obesity in 2022–2027, according to a decrease in the probability of move up one BMI class and an increase in the probability of move down one BMI class. Status-quo scenario corresponds to point ● at coordinates (0,0), scenario 1 ■ at (0, 22%), scenario 2 ◆ at (33%, 0), and scenario 3 ▲ at (33%, 22%). Scenarios 1 and 2 were built up to obtain a stabilization of the overall obesity prevalence: scenario 1 corresponds to a 22% decrease in the probability of move up one BMI class, and scenario 2 corresponds to a 33% increase in the probability of move down one BMI class. Scenario 3 corresponds to the combination of scenarios 1 and 2. The black line corresponds to the French government’s objective of 15%-reduction of overall obesity, for example: 98% of decrease in the probability of move up one BMI class alone, or 155% of increase in the probability of move down one BMI class alone.

In the status quo scenario, the estimated prevalence of overall obesity will increase from 17.3% in 2022 to 18.2% in 2027, i.e. 5.2% relative increase (Fig 5A). Fig 6 presents the dual effect of the decrease in the probability of move up one BMI class and the increase in the probability of move down one BMI class on the prevalence of obesity in 2027. On one hand, to achieve the French government’s objective of 15%-reduction of the prevalence of overall obesity, at least 98% of decrease in the probability of move up one BMI class or 155% of increase in the probability of move down one BMI class would be necessary, which appears unrealistic (Fig 6).

On the other hand, obtain a stabilization of the prevalence of overall obesity at 17.3% over 2022–2027 would require either a 22% decrease in the probability of move up one BMI class (scenario 1) or an in increase in the probability of move down one BMI class of 33% (scenario 2) (Fig 6). Consequently, the impact of a strategy based on the decrease in the probability of move up one BMI class (scenario 1) is higher than a strategy based on the increase in the the probability of move down one BMI class (scenario 2). Scenario 3 which combines scenarios 1 and 2 has a greater impact compared to either strategy alone and would require less effort for each strategy (Fig 6). Scenario 3 would lead to decrease the prevalence of overall obesity from 17.3% in 2022 to 16.4% in 2027, i.e. a relative decrease of 9.9% (Fig 5 and Table 2).

**Table 2:** Prevalence of overweight, obesity I, obesity II, obesity III and type 2 diabetes, from 2022 to 2027, according to status quo and the 3 scenarios. Scenarios 1 and 2 were built up to obtain a stabilization of the overall obesity prevalence: scenario 1 corresponds to a 22% decrease in the probability of move up one BMI class, and scenario 2 corresponds to a 33% increase in the probability of move down one BMI class. Scenario 3 corresponds to the combination of scenarios 1 and 2.

	2022	2023	2024	2025	2026	2027
**Overweight**						
Status-quo	31.6%	31.6%	31.6%	31.6%	31.7%	31.7%
Scenario 1	31.6%	31.6%	31.5%	31.5%	31.4%	31.2%
Scenario 2	31.6%	31.6%	31.6%	31.5%	31.4%	31.4%
Scenario 3	31.6%	31.5%	31.5%	31.3%	31.1%	30.9%
**Obesity I**						
Status-quo	11.8%	11.8%	11.9%	12.0%	12.1%	12.2%
Scenario 1	11.8%	11.8%	11.8%	11.8%	11.8%	11.7%
Scenario 2	11.8%	11.8%	11.8%	11.8%	11.8%	11.8%
Scenario 3	11.8%	11.8%	11.7%	11.6%	11.4%	11.2%
**Obesity II**						
Status-quo	4.1%	4.1%	4.2%	4.3%	4.3%	4.4%
Scenario 1	4.1%	4.3%	4.4%	4.5%	4.6%	4.6%
Scenario 2	4.1%	4.3%	4.4%	4.5%	4.6%	4.6%
Scenario 3	4.1%	4.2%	4.4%	4.4%	4.4%	4.3%
**Obesity III**						
Status-quo	1.5%	1.5%	1.6%	1.6%	1.7%	1.7%
Scenario 1	1.5%	1.4%	1.3%	1.2%	1.1%	1.0%
Scenario 2	1.5%	1.4%	1.2%	1.1%	1.0%	0.9%
Scenario 3	1.5%	1.4%	1.2%	1.1%	1.0%	0.9%
**Type 2 diabetes**						
Status-quo	7.3%	7.5%	7.7%	7.8%	8.0%	8.1%
Scenario 1	7.3%	7.5%	7.7%	7.8%	8.0%	8.1%
Scenario 2	7.3%	7.5%	7.7%	7.8%	8.0%	8.1%
Scenario 3	7.3%	7.5%	7.7%	7.8%	7.9%	8.1%

Table 2 provides the annual relative variation of prevalences of overweight, obesity I-III over the 5-year period compared to 2022, according to the status quo and scenarios 1 to 3. In the status quo, the prevalence of obesity III could increase from 1.5% in 2022 to 1.7% in 2027 (+13.3%) (Table 2 and Fig 5B). However, this prevalence could decrease to 0.9%-1.0% in 2030 if scenarios 1 to 3 were applied, corresponding to a relative reduction of at least 33.3% (Table 2 and Fig 5B). Moreover, the prevalence of obesity III shows early change, from the first year whichever the tested scenario (Table 2). On the opposite, the impact of the three scenarios on the other indicators (prevalence of overweight, obesity I-II) is less pronounced, early or at 5 years, and not visible for all scenarios (Table 2). Thus, a change in the prevalence of obesity III during the first two years could be an early indicator of the success or failure of a public health policy at 5 years (Fig 5B and Table 2): an increase in obesity III prevalence during the first 2 years would indicate failure while a decrease would indicate success. As an example, scenario 1 is associated with a decrease of obesity III of 6.7% at one year and 13.3% at two years (respectively, -6.7% and -13.3% for scenarios 2 and 3).

### Model’s predictions of type 2 diabetes prevalence and lifetime risk

In the status quo scenario, the estimated prevalence of type 2 diabetes will increase from 7.3% in 2022 to 8.1% in 2027, i.e. 9.9% relative increase (Table 2). Regardless of the scenario, the estimated prevalence of type 2 diabetes would continue to increase to 8.1% even in the scenarios with decreasing obesity (Table 2).

The lifetime cumulative risk of developing type 2 diabetes for different 25 year-old individuals in 2022, depending on their gender, BMI category at baseline and its evolution over time is illustrated on S1 Fig. For example, men (vs. women) without type 2 diabetes and with normal weight (at risk of being overweight or obese) would have a lifetime risk of developing type 2 diabetes of 30.9% (vs. 28.3%) whereas when remaining in normal weight all their life, their lifetime risk of developing type 2 diabetes would be 8.9% (vs. 8.5%). When obese class I, II or III at baseline, the lifetime risk of developing type 2 diabetes would be increased. All scenarios have a great impact on lifetime risk of type 2 diabetes (S2 Table).

**Fig 7:**
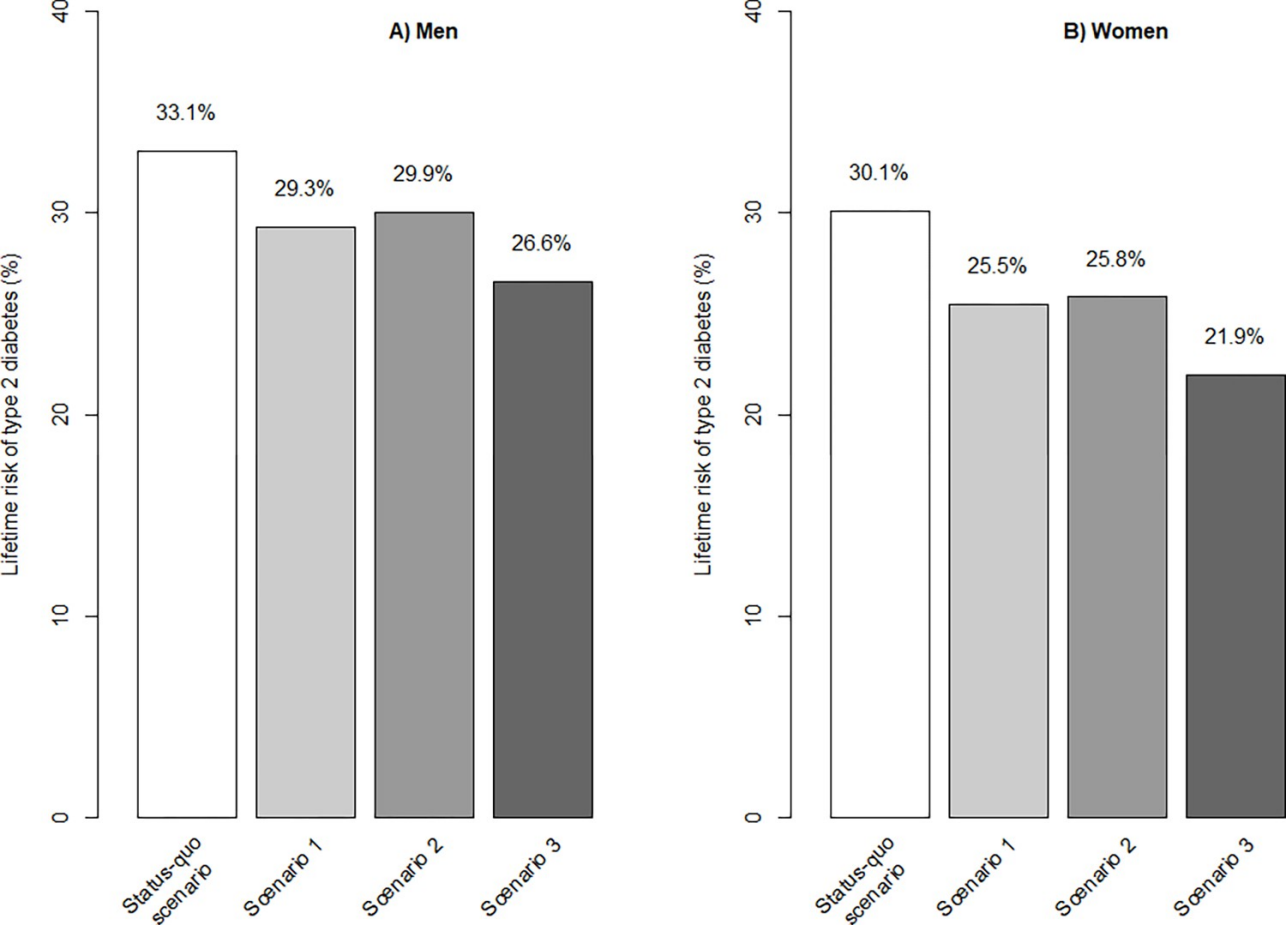
Lifetime risk of developing type 2 diabetes, for cohorts of 25 years-old individuals according to scenarios. Initial weight distribution is representative of the one of the 25 years-old individuals in 2022: A) men, 73.2% normal weight, 17.9% overweight, 6.6% obese class I, 1.9% obese class II, 0.5% obese class III, and B) women, 81.0% normal weight, 9.7% overweight, 5.4% obese class I, 2.6% obese class II, 1.3% obese class III. Scenarios 1 and 2 were built up to obtain a stabilization of the overall obesity prevalence: scenario 1 corresponds to a 22% decrease in the probability of move up one BMI class, and scenario 2 corresponds to a 33% increase in the probability of move down one BMI class. Scenario 3 corresponds to the combination of scenarios 1 and 2.

Moreover, the model projected the distribution of BMI categories of 25-year old individuals according to gender, in 2022: 73.2% normal weight, 17.9% overweight, 6.6% obese class I, 1.9% obese class II, 0.5% obese class III for men, and 81.0% normal weight, 9.7% overweight, 5.4% obese class I, 2.6% obese class II, 1.3% obese class III for women. Taking into account these distributions, Fig 7 presents the lifetime risk of type 2 diabetes of those men and women according to the status quo scenario and the three alternative scenarios: stabilizing the prevalence of obesity would allow to decrease the lifetime risk of type 2 diabetes by 11.5% (from 33.1% to 29.3% in scenario 1) and 9.4% (from 33.1% to 30.0% in scenario 2) in men, and by 15.3%, (from 30.1% to 25.5% in scenario 1) and 14.3% (from 30.1% to 25.5 in scenario 2), in women. Scenario 3 would decrease lifetime risk of type 2 diabetes by 19.6% in men (from 33.1% to 26.6%) and 27.2% in women (from 30.1% to 21.9%).

## Discussion

In the present study, a Markov model was developed based on 97,948 individual French data to predict the progression of becoming overweight, obesity and type 2 diabetes over time. This model showed that the prevalence of overall obesity and type 2 diabetes is likely to continue to increase, in the status quo scenario, from 17.3% to 18.2%, and from 7.3% to 8.1%, respectively, between 2022 and 2027. The overall lifetime risk of diabetes in men and women aged 25 in 2022 is 33.1% and 30.1%, respectively. Our model is useful for ability to estimate the impact of different public health policies on the burden of obesity and type 2 diabetes. It showed that a strategy to prevent the probability of move up one BMI class in all individuals would be more effective than promoting the probability of move down one BMI class in overweight and obese individuals. It showed that prevalence of obesity III would be an efficient surrogate marker for the early prediction of the success or failure of the public health strategy. Finally, the model showed that public health policies stabilizing or reducing obesity would not influence the prevalence of type 2 diabetes at 5 years, but would decrease the lifetime risk of type 2 diabetes.

Understanding the dynamics of the burden of obesity and type 2 diabetes is a complex issue that can be evaluated by a modelling approach that takes into account cofactors such as sex and age as well as the relationship between the risk of type 2 diabetes and becoming overweight or the development of obesity. Sex and age are double-sided factors. All other things being equal, female gender has a protective effect on becoming overweight (HR = 0.72) and developing type 2 diabetes (HR = 0.81) but is an aggravating factor for risk of obesity once being overweight (HR = 2.00). Also, individuals over age 50 have higher risks of progressing from normal weight to overweight or of developing type 2 diabetes, and a lower risk of progressing from being overweight to obesity. One explanation for these dualities could be explain by differences in hormonal profiles and environemental factors according to gender and age [25]. BMI has a major impact on the risk of type 2 diabetes that is increased by 17 times between individuals in normal weight (who have a quasi nul annual risk = 0.09%) and individuals in obesity II or III (annual risk = 1.56%). This increase is in agreement with previous studies on the relationship between BMI and type 2 diabetes [12, 26–29].

Despite various national plans to fight the burden of overall obesity, its prevalence has steadily increased and, according to the prediction model, it will continue to do so in the next 5 years. However, we cannot exclude that these different plans could have slowed down the increase in the prevalence of overall obesity. If the success of a public health policy is at least to stabilize the prevalence of overall obesity, the progression rates from one BMI category to the next (move up one BMI class) should be decreased by at least 22% or the regression from one BMI category to the previous category (move down one BMI class) should be increased by 33%. This implies for example that the transition rate of progression from normal weight to overweight would be 3.74% (vs. 4.80%) and from overweight to obesity I, from obesity I to obesity II and from obesity II to obesity III would be 0.98% (vs. 1.25%).

Our model showed that health policies to stabilize or reduce obesity would not decrease the prevalence of type 2 diabetes over 5 years. Indeed, a decrease in type 2 diabetes has only been described in studies with intensive lifestyle changes, rather than in the general population, and these changes rarely persist during long-term follow-up [12]. Although this could suggest that public health policies to reduce obesity will not affect type 2 diabetes, it will nevertheless reduce the lifetime risk of type 2 diabetes. Indeed, our model found that a health policy stabilizing overall obesity would decrease the lifetime risk of type 2 diabetes by 13.6% in overweight individuals, 9.8% in obese I, 5.8% in obese II and 3.2% in obese III (Scenario 1, 25 y-o men; S2 Table). Our results are in accordance with a previous study focusing on lifetime risk of diabetes [30], that could only be identified using a model.

Worldwide public health goals focus on limiting weight gain in the general population. These strategies led to a reduction in the number of new cases of overweight, obese and diabetes individuals. The model showed that strategies reducing progression from one BMI category to the next are more efficient than those increasing regression from one BMI category to the previous category. In the present study we observed that the goal of the French Ministry of health of a 15% decrease in the prevalence of obesity will be difficult too reach when considering the required decrease in the probability of move up one BMI class or increase in the probability of move down one BMI class. Thus, combining move up and move down one BMI class strategies is more effective than each strategy alone. It should be also noted that only move down one BMI class strategies can target individuals that are already overweight, obese and/or diabetic.

The effect on overall obesity of scenarios that influence transition rates between BMI categories (move up or down one BMI class) by around 30% will stabilize or slightly reduce obesity at 5 years. More benefit is seen with these scenarios after 10 years (S3 Table). However 10 years is not adapted to policy makers who cannot wait this long to adjust their strategy. It is interesting to note that the prevalence of obesity III is an early predictor. For example scenario 1 stabilizes overall obesity at 5 years and results in a early decrease in the prevalence of obesity III: -6.7% at 1 year and -13.3% at 2 years (Table 2). Surveillance of obesity III could be an effective surrogate marker for the early assessment of the long-term progression of obesity and to early identify a successful public health policy. Such tool may be particularly useful in countries with a severe obesity epidemic such as in the United States.

Our study has certain limitations. First, the model was based on survey data with self-declared weights, that may have under-evaluated participants’ weight. However, the prevalence of overweight individuals and obesity was confirmed in two different repeated surveys (ESPS, Obépi-Roche) and one study that objectively determined weight and height during the consultation [31]. Second, our model assumed that all participants were at-risk of progressing across BMI categories as of 3 years-old and at-risk of developing type 2 diabetes after 25 years-old in overweight and obese individuals and at 40 years-old in normal weight individuals. Indeed, these assumptions were based on the International Obesity Task Force stating that overweight and obesity cannot be defined before the age of 2, and because data collected in the ESPS surveys shows no type 2 diabetes before these ages. Thirdly, we do not have data in children under 15 years-old since they were not included in the ESPS surveys. As observed in the surveys, overweight and obesity were already present in some individuals aged 15, meaning that the development of this event(s) occurred during childhood or puberty. Although we agree that progression/regression between BMI categories may differ in the under 25 age group, the estimated rates were an average of the different periods (3–14 years-old, 15–24 years-old). This approach was realistic since the fit of the model was good. For example, it predicted that in 2014, 6.1% of the 15-24-year olds would be obese, which is close to the prevalence observed in the survey (5.4%). In addition, we only had 5,505 minor respondents (5.6% of the 97,948 included) which was insufficient to define an age group of 15 to 17 years. As the fit of the model was only evaluated in individuals aged 15 years and older, this model should only be used in this population.

Lastly, although bariatric surgery is benefit for long-term diabetes remission [14, 16], regression of diabetes was not considered. Indeed, we had no information about who undergone bariatric surgery in the ESPS surveys. As an example, in 2014, this could have concerned 2.6% of the French individuals in obesity class II or III (55,000 procedures / 2,100,000 obese II or III) and therefore represents for only 0.1% of diabetes remission among diabetics in 2014 (2.6% x 16% diabetics x 30% diabetes remission) [32–35]. Therefore, this limit would not have a significant impact on our results.

## Conclusion

The present model provides novel insights in the prediction of obesity, overall and by class, and type 2 diabetes, to help health policy makers develop their strategy and anticipate their future impact. This model identifies the evolution of obesity III as an early indicator of success of public health policy. Public health policies stabilizing or decreasing overall obesity may lead to decrease lifetime risks of type 2 diabetes without affecting short-term type 2 diabetes prevalence.

## Supporting information

S1 Appendix Background mortality. (DOCX)

S1 Table Characteristics of included respondents from the ESPS database. (DOCX)

S2 Table Lifetemine risk of developing type 2 diabetes for cohorts of 25 years-old individuals in 2022, depending on sex, initial BMI of cohort, and scenario. Scenarios 1 and 2 were built up to obtain a stabilization of the overall obesity prevalence: scenario 1 corresponds to a 22% decrease in the probability of move up one BMI class, and scenario 2 corresponds to a 33% increase in the probability of move down one BMI class. Scenario 3 corresponds to the combination of scenarios 1 and 2. We compare here individuals who remain in the normal weight state all life long, and those who may gain and/or lose weight as assessed by the model.
(DOCX)

S3 Table Relative variation between prevalence of obesity in 2022 and those predicted at 5 (2027) and 10 (2032) years according to scenarios. Scenarios 1 and 2 were built up to obtain a stabilization of the overall obesity prevalence: scenario 1 corresponds to a 22% decrease in the probability of move up one BMI class, and scenario 2 corresponds to a 33% increase in the probability of move down one BMI class. Scenario 3 corresponds to the combination of scenarios 1 and 2.
(DOCX)

S1 Fig Cumulative lifetime risk of developing type 2 diabetes, for 25 years-old inviduals in 2022, depending on their initial BMI category: A) men and B) women. We compare here individuals who remain in the normal weight state all life long, and those who may gain and/or lose weight as assessed by the model.
(TIF)
