# Supplementary material for: Early prediction of the impact of public health policies on obesity and lifetime risk of type 2 diabetes: A modelling approach
Source: PLoS One. 2024 Mar 28;19(3):e0301463. doi: 10.1371/journal.pone.0301463 (PMC10977742; doi:10.1371/journal.pone.0301463)
Supplement: S1 Appendix — (DOCX) [file pone.0301463.s001.docx]

# **Appendix accompanying the manuscript titled “Early prediction of the impact of public health policies on obesity and lifetime risk of type 2 diabetes: a modelling approach”**

Pierre Bauvin*, Claire Delacôte*, Line Carolle Ntandja Wandji,Guillaume Lassailly, Violeta Raverdy, François Pattou, Sylvie Deuffic-Burban**, Philippe Mathurin**

*equal contribution (co-first authors), **equal contribution (co-last and -corresponding authors)

# BACKGROUND MORTALITY

We used the French mortality tables to estimate the risk of overall mortality for every age, sex and year in the general population. The transition rates were multiplied by hazard ratios of overall mortality for every BMI category and age. These hazard ratios were recalculated from the work of the Global BMI Mortality Collaboration (1). Indeed, the hazard ratios of overall death in Europe, were provided compared to normal weight. Thus, they had to be re-weighted to obtain hazard ratios of overall death versus the general population. They were then linearly interpolated to provide continuous hazard ratio according to age. For example, hazard ratios of overall mortality for normal weight patients, versus the general population, vary from 0.84 to 1.00, depending on age, whereas for overweight patients, they vary from 0.95 to 1.00. For obesity I, they vary from 1.08 to 1.59; for obesity II, from 1.51 to 2.52 and for obesity III, from 1.85 to 4.47.

Moreover, we used additional risks factor accounting for the effect of type 2 diabetes on overall mortality, the Regidor et al’s hazard ratios of mortality in type 2 diabetes versus non- type 2 diabetes, adjusted for age, sex and obesity (2). Similarly to above, these hazard ratios were re-weighted from the original article, in order to result in hazard ratios of overall death versus the general population.

# REFERENCES

1. Global BMI Mortality Collaboration, Di Angelantonio E, Bhupathiraju Sh N, et al. Body-mass index and all-cause mortality: individual-participant-data meta-analysis of 239 prospective studies in four continents. *Lancet*. 2016;388(10046):776-86.

2. Regidor E, Franch J, Segui M, Serrano R, Rodriguez-Artalejo F, Artola S. Traditional risk factors alone could not explain the excess mortality in patients with diabetes: a national cohort study of older Spanish adults. *Diabetes Care*. 2012;35(12):2503-9.
