# Supplementary material for: Early prediction of the impact of public health policies on obesity and lifetime risk of type 2 diabetes: A modelling approach
Source: PLoS One. 2024 Mar 28;19(3):e0301463. doi: 10.1371/journal.pone.0301463 (PMC10977742; doi:10.1371/journal.pone.0301463)
Supplement: S1 Table — (DOCX) [file pone.0301463.s002.docx]

|  | **All surveys** |
| --- | --- |
| **Number of respondents, n** | 97,948 |
| **Age, mean (SD)** | 44.7 (18.7) |
| **Male sex, n (%)** | 50,426 (51.5) |
| **BMI (kg/m²), mean (SD)** | 24.6 (5.3) |
| **BMI category, n (%)** |  |
| **Normal weight or underweight (BMI < 25)** | 59,257 (60.5) |
| **Overweight (25 ≤ BMI < 30)** | 27,573 (28.2) |
| **Obesity class I (30 ≤ BMI < 35)** | 8,424 (8.6) |
| **Obesity class II (35 ≤ BMI < 40)** | 2,007 (2.0) |
| **Obesity class III (BMI ≥ 40)** | 687 (0.7) |
| **Type 2 diabetes, n (%)** | 3,741 (3.8) |
