# Supplementary material for: Early prediction of the impact of public health policies on obesity and lifetime risk of type 2 diabetes: A modelling approach
Source: PLoS One. 2024 Mar 28;19(3):e0301463. doi: 10.1371/journal.pone.0301463 (PMC10977742; doi:10.1371/journal.pone.0301463)
Supplement: S2 Table — Scenarios 1 and 2 were built up to obtain a stabilization of the overall obesity prevalence: scenario 1 corresponds to a 22% decrease in the probability of move up one BMI class, and scenario 2 corresponds to a 33% increase in the probability of move down one BMI class. Scenario 3 corresponds to the combination of scenarios 1 and 2. We compare here individuals who remain in the normal weight state all life long, and those who may gain and/or lose weight as assessed by the model. (DOCX) [file pone.0301463.s003.docx]

| **Sex** | **Initial BMI** | **Status-quo** | **Scenario 1** | **Scenario 2** | **Scenario 3** |
| --- | --- | --- | --- | --- | --- |
| Women | Normal remaining weight | 8.5% | 8.5% | 8.5% | 8.5% |
|  | Normal weight (with BMI progression) | 28.3% | 22.0% | 24.8% | 19.4% |
|  | Overweight | 34.1% | 27.7% | 29.1% | 23.5% |
|  | Obese I | 38.8% | 32.8% | 32.9% | 27.5% |
|  | Obese II | 43.2% | 38.1% | 36.8% | 32.0% |
|  | Obese III | 47.6% | 43.4% | 40.6% | 36.4% |
| Men | Normal remaining weight | 8.9% | 8.9% | 8.9% | 8.9% |
|  | Normal weight (with BMI progression) | 30.9% | 25.5% | 28.4% | 23.5% |
|  | Overweight | 36.0% | 31.0% | 32.7% | 28.0% |
|  | Obese I | 42.7% | 38.5% | 38.3% | 34.2% |
|  | Obese II | 49.9% | 47.0% | 44.9% | 41.8% |
|  | Obese III | 56.5% | 54.7% | 51.2% | 49.1% |
