# Supplementary material for: Early prediction of the impact of public health policies on obesity and lifetime risk of type 2 diabetes: A modelling approach
Source: PLoS One. 2024 Mar 28;19(3):e0301463. doi: 10.1371/journal.pone.0301463 (PMC10977742; doi:10.1371/journal.pone.0301463)
Supplement: S3 Table — Scenarios 1 and 2 were built up to obtain a stabilization of the overall obesity prevalence: scenario 1 corresponds to a 22% decrease in the probability of move up one BMI class, and scenario 2 corresponds to a 33% increase in the probability of move down one BMI class. Scenario 3 corresponds to the combination of scenarios 1 and 2. (DOCX) [file pone.0301463.s004.docx]

|  | At 5 years (2027) | At 10 years (2032) |
| --- | --- | --- |
| Status-quo | +5.3% | +9.3% |
| Scenario 1 | +0.0% | -4.1% |
| Scenario 2 | +0.0% | -3.5% |
| Scenario 3 | -5.2% | -15.5% |
