# Supplementary figures and images for: Early prediction of the impact of public health policies on obesity and lifetime risk of type 2 diabetes: A modelling approach
Source: PLoS One. 2024 Mar 28;19(3):e0301463. doi: 10.1371/journal.pone.0301463 (PMC10977742; doi:10.1371/journal.pone.0301463)

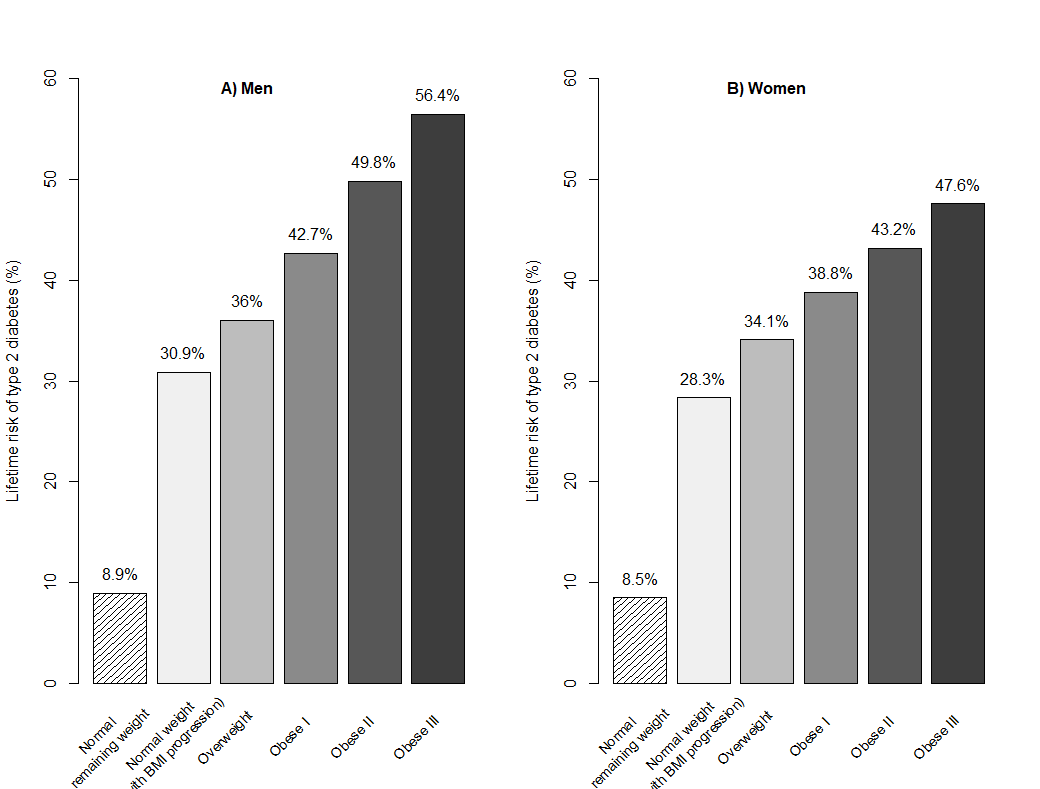

Supplement: S1 Fig — Cumulative lifetime risk of developing type 2 diabetes, for 25 years-old inviduals in 2022, depending on their initial BMI category: A) men and B) women. We compare here individuals who remain in the normal weight state all life long, and those who may gain and/or lose weight as assessed by the model. (TIF) [file pone.0301463.s005.tif]
